# Improving Use of Social Communicative Gestures by Children with Autism

**DOI:** 10.3390/bs16030401

**Published:** 2026-03-10

**Authors:** Rebecca J. Barall, M. Alice Shillingsburg

**Affiliations:** Integrated Center for Autism Spectrum Disorders (iCASD), Munroe-Meyer Institute for Genetics and Rehabilitation, University of Nebraska Medical Center, Omaha, NE 68106, USA; ashillingsburg@unmc.edu

**Keywords:** gestures, pointing, social communication, social skills, autism spectrum disorder

## Abstract

Difficulties in social communication are a core characteristic of autism. Gesture use in children with autism is often delayed or atypical, with reduced frequency, diversity, and spontaneity. Pointing gestures, which typically emerge between 9 and 12 months of age, have been shown repeatedly to predict later language acquisition in both neurotypically developing children and those with autism. Thus, the deficits in proximal and distal pointing gestures observed in children with autism may impede social communication and language learning. Employing a nonconcurrent multiple baseline across participants design, this study examined the efficacy of prompting and reinforcement for teaching proximal pointing to request in 12 children with autism, aged 3 to 11 years. Results showed that 9 of the participants acquired proximal pointing and subsequently emitted distal pointing at distances of 0.61 m, 1.22 m, and 1.83 m (2, 4, and 6 feet) without additional intervention. Proximal and distal pointing was maintained at 4-week follow-up. However, not all participants acquired proximal pointing, highlighting potential variability related to individual characteristics and the need for modified procedures. These findings provide support for the use of prompting and reinforcement to teach socially communicative gestures in children with autism.

## 1. Introduction

### 1.1. Gesture Development and Taxonomy

Children typically begin producing gestures between 8 and 12 months of age (Bates et al., 1975), before producing spoken language. These early gestures provide a critical foundation for social communication and are reliable predictors of later language outcomes (e.g., Acredolo & Goodwyn, 1988; Iverson & Goldin-Meadow, 2005; Rowe et al., 2008, Rowe & Goldin-Meadow, 2009). For example, a child’s ability to point to an object increases the likelihood of learning the corresponding word within the following months. Gesture and spoken language follow an interdependent developmental trajectory, and delays in one are often accompanied by corresponding delays in the other (Goldin-Meadow, 2009). The relationship between gesture and language development highlights the potential benefits of targeted interventions aimed at promoting gesture use. Experimentally inducing gesture use in infants has been shown to facilitate early spoken word development (e.g., LeBarton et al., 2015). Moreover, when vocal speech is ambiguous or difficult to interpret, the presence of gestures can enhance listener understanding (Goldin-Meadow & Alibali, 2013).

Gestures can be grouped into three primary types: *deictic* gestures that direct attention to objects, people, or locations; *representational* gestures that symbolize characteristics or actions of objects; and *conventional* gestures that follow culturally standardized meanings (e.g., Novack & Goldin-Meadow, 2017). This framework differentiates gesture types based on their defining features. Deictic gestures include pointing, reaching, showing, and giving, and are among the earliest gestures used by children to direct attention to specific objects or events in their environment (Behne et al., 2012; Iverson & Thal, 1998). In contrast to representational or conventional gestures, the meaning of deictic gestures depends on the environmental context in which the gesture occurs rather than on its physical form (Goldin-Meadow & Alibali, 2013).

Some children, particularly those with developmental disabilities, may also produce idiosyncratic or unconventional gestures, sometimes called *indicating responses.* These gestures communicate intent but do not conform to culturally recognized forms, such as pointing or giving (Frampton et al., 2024; Sigafoos et al., 2000). For example, a child may tap a caregiver’s hand or push an object toward the caregiver to request it. Although these gestures convey communicative intent, they differ from conventional or deictic gestures in form and may be less reliably interpreted by others.

#### Pointing

The present study focuses on pointing, a critical deictic gesture, due to its pivotal role in early communicative development. *Imperative* pointing is emitted to request to obtain a desired object and can be conceptualized in behavior–analytic terms as a mand (Skinner, 1957), a behavior under the functional control of an establishing operation (EO) and maintained by access to a specific reinforcer (Laraway et al., 2003). The presence of a desired object serves as an EO, increasing the likelihood that the child will point, and the person delivering the object serves as a reinforcing consequence. Imperative pointing can further be classified into *proximal* (involving contact or close proximity to the object, typically appearing between 9 and 13 months) and *distal* (directed toward objects at a distance without physical contact, typically emerging in the second year of life; Carpenter et al., 1998). Research suggests that children diagnosed with autism often show impairments in both proximal and distal pointing (e.g., Toth et al., 2007; Mishra et al., 2021).

Whereas imperative pointing is used to initiate requests, *declarative pointing* is used to share interest or direct another person’s attention to an object or event. From a behavior–analytic perspective, declarative pointing functions as a tact (Skinner, 1957) to label a referent, with the social attention or shared gaze of the adult serving as the reinforcing consequence. The child emits the point to gain shared social reinforcement, such as adult attention, rather than to obtain a tangible object.

As a gesture that directs attention, the development of pointing is closely linked with joint attention (JA), a core social–communicative skill involving shared attention between individuals. JA typically includes a combination of eye gaze alternation, gestures, and vocalizations. JA develops through social reinforcement, with caregivers’ responses acting as contingent reinforcers that shape pointing behavior (e.g., Dube et al., 2004; Michael, 1982). From a behavioral perspective, antecedent events, such as the presence of an engaging object, can increase the likelihood of pointing by enhancing the reinforcing value of adult responses, like shared gaze or attention.

Pointing often initiates JA and functions as an early form of social referencing, in which the child’s behavior is guided by discriminative properties of the caregiver’s facial, vocal, or gestural responses. Social referencing can be conceptualized as a behavior chain in which an object or event prompts the child to shift gaze toward the caregiver, whose response then serves as a discriminative stimulus that informs the child’s subsequent behavior, such as approaching, avoiding, or further indicating (Pelaez et al., 2012). These caregiver responses can acquire reinforcing value, strengthening both gaze shifting and pointing behavior, and supporting the establishment of socially mediated contingencies. For example, when a child points to an object, a caregiver may label the referent while providing the desired item, thereby creating a contingent, reciprocal interaction that reinforces social engagement and word learning (Özçalışkan & Dimitrova, 2013). This relationship between pointing and JA further underscores the role of environmental contingencies and discriminative stimuli in shaping early social–communicative gestures and provides rationale for interventions targeting pointing, particularly for individuals who may demonstrate deficits in JA and social referencing.

### 1.2. Children with Autism and Gestures

Autism spectrum disorder (ASD) is a neurodevelopmental condition characterized by difficulties in social communication and restricted, repetitive behaviors (American Psychiatric Association, 2022). Children with ASD^1^ exhibit substantial variability in the development of communication and language skills, and frequently display an atypical joint attention profile, characterized by reduced frequency and quality of shared attention behaviors (e.g., Chawarska et al., 2007). Approximately 1 in 31 eight-year-old children in the U.S. are diagnosed with ASD (Shaw et al., 2025), and about 65% of those may experience moderate to severe language delays (Anderson et al., 2007). Among children with ASD, an estimated 20–30% remain minimally verbal or non-speaking throughout the lifespan, often producing few functional words and relying on limited or idiosyncratic gestures to communicate (Rose et al., 2016; DiStefano & Kasari, 2016; Kasari et al., 2013). These communication challenges highlight the importance of early interventions targeting social–communicative skills, as research suggests that the most optimal period for acquiring spoken language in children with ASD generally occurs before eight years of age (Bal et al., 2019; Wodka et al., 2013).

Differences in gesture use between infants with ASD and typically developing (TD) children can be observed as early as 9 to 12 months, with TD infants showing more rapid increases in both the frequency and variety of gestures, whereas infants later diagnosed with ASD often demonstrate significant delays and overall reduced, less diverse, and less spontaneous gesture use (e.g., Watson et al., 2013). These early gesture delays may have particularly important implications for minimally verbal children with ASD, as reduced frequency and spontaneity of deictic gestures, such as pointing, may limit caregiver responsiveness and reduce opportunities for language learning and social engagement. Minimally verbal children may rely on indicating responses, defined as rudimentary nonverbal behaviors that communicate intent but do not resemble conventional gestures (Frampton et al., 2024; Sigafoos et al., 2000). The use of unconventional or idiosyncratic gestures rather than universally recognized gestures, such as pointing, giving, showing, or reaching, can negatively impact the quality of social communication and the development of effective communication, particularly in children who are minimally verbal or nonspeaking (Sigafoos et al., 2000; Wetherby et al., 2007). Delays and atypical patterns in gesture use are particularly concerning for minimally verbal children with ASD because gestures, like spoken words, can predict later communication abilities (Choi & Rowe, 2025; Özçalışkan et al., 2016).

Although research on gesture development in ASD has recently increased, most studies have focused on infants at elevated likelihood of autism but not yet diagnosed or on verbally fluent children with autism (e.g., Choi et al., 2021; Mishra et al., 2021). There are significant gaps in understanding how children with ASD, particularly those who are minimally verbal, develop communication skills and respond to interventions aimed at improving language outcomes (Tager-Flusberg & Kasari, 2013). Early, targeted interventions that explicitly teach social communicative gestures, particularly pointing, may provide a critical foundation for subsequent language development for children with ASD.

### 1.3. The Present Study

Despite the established role of gestures in language acquisition, there is a notable gap in research examining the efficacy of experimentally inducing the acquisition of pointing in children with ASD. Moreover, it is unknown whether the acquisition of proximal pointing leads to generalization to distal pointing and whether these skills are maintained over time. Thus, the present study evaluated the effectiveness of a treatment designed to teach imperative proximal pointing to children with ASD.

We addressed three research questions: (1) Do children with ASD acquire proximal pointing following intervention? (2) If proximal pointing is acquired, does pointing generalize to increasing distal distances of 0.61 m, 1.22 m, and 1.83 m (2, 4, and 6 feet) without additional intervention? (3) Is pointing maintained over time at both proximal and distal distances, as assessed at a 4-week follow-up?

## 2. Methods

### 2.1. Participants, Setting, and Materials

The study was conducted with 12 participants diagnosed with ASD between the ages of 3 years, 0 months and 11 years, 11 months. Participant characteristics are presented in Table 1. All participants received applied behavior analysis (ABA) services at a clinic within a university-based medical center. During the study, participants received ABA services a mean of 4.8 days per week (range: 4–5 days) for a mean of 3.5 h per day (range: 3.17–4.25 h). Each participant received the intervention as part of their clinical program for an average of 4–8 weeks. No participants were prospectively recruited or enrolled for research purposes. We conducted a retrospective review of medical records to obtain demographic and assessment data for participants who received the intervention as part of standard clinical care between 1 January 2025 and 19 October 2025. The study was approved by the institutional review board as a medical record review protocol (IRB #0739-25-EP).

Participants were included in the study if they (a) had a documented diagnosis of ASD; (b) had significant impairments in social communication and expressive vocal communication; (c) exhibited limited gesture use, specifically limited production of pointing (e.g., to request or show objects); and (d) had received the packaged pointing intervention (described below; Section 2.6) in as part of their care. Each participant’s individualized program included goals targeting expressive communication development, which were regularly reviewed in collaboration with caregivers.

All sessions for each participant were conducted in the same designated classroom and instructional area. Sessions occurred in either a large classroom (4 × 10 m) or a small classroom instructional area (3 × 3 m), each containing one table and two chairs. Preferred items, as nominated by each participant’s clinical team, were available at the table and varied by participant (e.g., animal figurines, bubbles, musical toys, or edible snacks). Materials also included paper data sheets, writing utensils, a tape measure to record the distance from the participant to stimuli, and non-toy stimuli presumed to be neutral or non-preferred by participants (e.g., office supplies).

The *Verbal Behavior Milestones Assessment and Placement Program* (VB-MAPP; Sundberg, 2008) is an assessment tool used to identify an individual’s current language skills across developmental milestones, organized into three age levels: 0–18 months, 18–30 months, and 30–48 months. The VB-MAPP was administered to participants as part of their routine clinical services prior to intervention (see Table 1 for VB-MAPP domain scores). These scores were obtained from participants’ medical records in accordance with an IRB-approved medical record review protocol; therefore, direct measures of procedural integrity and reliability specific to the assessment administrations were not available as part of the current study.

Results indicated that participants exhibited limited manding skills (i.e., requests; Skinner, 1957); reduced independence in social engagement including joint attention behaviors; and significant impairments in sound production, vocal imitation, and the functional use of vocalizations for communication. Across participants, overall reported VB-MAPP milestone scores ranged from 10 to 109.5 points (out of 170), primarily spanning developmental levels from Level 1 (0–18 months developmental equivalence) to mid-to-late Level 2 (18–30 months developmental equivalence). Gavin was the only participant whose VB-MAPP scores showed emerging Level 3 (30–48 months developmental equivalence) skills. For Nico, their VB-MAPP milestone score was reported as 13, corresponding to Level 1, although individual scores for the mand, social, and vocal domains were not reported.

### 2.2. Design

This study employed a single-case experimental design (SCED) using a multiple baseline (MBL) approach across participants. In a standard MBL design, the introduction of an intervention is staggered across participants, behaviors, or settings to demonstrate experimental control without withdrawing treatment (Kazdin, 2019). This study used a nonconcurrent MBL design, in which participants began baseline at different time points rather than concurrently.

To clarify the logic of experimental control in a nonconcurrent MBL design, it may be useful to reference a traditional AB design, in which a participant’s behavior is measured during a baseline phase (A) and again after the introduction of an intervention (B; see Byiers et al., 2012 for an additional description of AB designs). Nonconcurrent MBL designs extend this same logic across multiple participants who begin baseline at different time points, allowing each participant to serve as their own control. This approach provides flexibility in recruitment and intervention timing while mitigating potential threats from history and maturation effects.

### 2.3. Dependent Variables

The primary dependent variable was the percentage of trials with independent proximal pointing, defined as pointing occurring within 0.30 m (12 inches) of the referent. The secondary dependent variable was the percentage of trials with independent distal pointing, defined as pointing occurring at a distance greater than 0.30 m, measured at 0.61 m, 1.22 m, and 1.83 m (2, 4, and 6 feet) for the current study. Operational definitions of proximal and distal pointing were adapted from Ramos-Cabo et al. (2021) and are presented in Table 2.

If the participant emitted an independent point at the specified distance, the researcher marked “I” for “independent.” An incorrect response was defined as the absence of an extended index finger point within five seconds of presenting the target stimulus, the emission of another indicating response (e.g., reaching, shifting eye gaze) without pointing, and/or the production of any other vocal or non-vocal behavior that did not meet the criteria for an index finger point. If an incorrect response occurred, the researcher marked “E” for “error” on the data sheet.

### 2.4. Assent Measures

Participant assent was measured both at the beginning of each session and continuously throughout the session. Table 2 presents operational definitions of assent and assent withdrawal, which were developed based on recommendations from C. Morris et al. (2021, 2024), and Breaux and Smith (2023). If assent was not given at the beginning of the session, the session was not initiated. If the participant assented to the session and continued to assent throughout, the experimenter marked “yes” on the data sheet. If assent was withdrawn during the session, the experimenter marked “no” on the data sheet. If the participant withdrew assent and did not re-assent following a break from the instructional area lasting up to 5 min for reassessment of motivation, the session was terminated, and data collection resumed at the next scheduled session.

### 2.5. Interobserver Agreement and Treatment Integrity

Trial-by-trial reliability was scored in vivo by a second trained observer using a separate data sheet (see Supplemental Information, Figure S1 for the data sheet used by both the primary and secondary observers). Interobserver agreement (IOA) for each trial was calculated by dividing the number of agreements by the total number of agreements and disagreements and multiplying by 100. Across all participants and all sessions, IOA data were collected for 32.09% of total trials, with a mean agreement of 99.42% (range: 80–100%). Broken down by participant, IOA was as follows across all phases: Luke, 75 of 255 trials (29%), 100% agreement; Virginia, 26 of 65 trials (40%), 100% agreement; Harry, 37 of 105 trials (35%), 98% agreement; Nico, 66 of 220 trials (30%), 99% agreement; Amber, 53 of 140 trials (38%), 100% agreement; Catherine, 50 of 150 trials (33%), 100% agreement; Gavin, 30 of 60 trials (50%), 97% agreement; Jasper, 56 of 175 trials (32%), 100% agreement; Hazel, 70 of 220 trials (32%), 100% agreement; Calvin, 73 of 250 trials (29%), 100% agreement; Esther, 71 of 235 trials (30%), 99% agreement; and Peyton, 75 of 250 trials (30%), 100% agreement.

Treatment integrity (TI), defined as adherence to the pointing intervention protocol steps, was also scored in vivo by a trained second observer for selected sessions. A TI checklist was developed (see Supplemental Information, Figure S2), and TI was calculated for each session by dividing the number of protocol steps delivered with fidelity by the total number of applicable steps and multiplying by 100 to yield a percentage. Across all participants and all sessions, TI data were collected for 43.30% of total sessions, with a mean integrity score of 100% across all participants. Broken down by participant, TI was as follows across all phases: Luke, 32 of 51 sessions (63%); Virginia, 5 of 13 sessions (38%); Harry, 9 of 21 sessions (43%); Nico, 18 of 44 sessions (41%); Amber, 11 of 28 sessions (39%); Catherine, 12 of 30 sessions (40%); Gavin, 6 of 12 sessions (50%); Jasper, 14 of 35 sessions (40%); Hazel, 18 of 44 sessions (41%); Calvin, 20 of 50 sessions (40%); Esther, 19 of 47 sessions (40%); and Peyton, 20 of 50 sessions (40%).

### 2.6. General Procedures for Packaged Pointing Intervention

Each participant completed intervention sessions between two and five days per week. Session blocks for each participant lasted between 15 and 30 min and included 3–6 individual sessions per block, with each session lasting approximately 5–10 min. Prior to each session block, the instructional area was arranged with preferred and neutral items. Preferred items were those that were nominated by each participant’s Board Certified Behavior Analyst (BCBA) and clinical staff as historically preferred and potentially motivating. Neutral non-toy items (e.g., office supplies) were also available to present in an array alongside the participant-identified target items. Presenting target and non-target items in an array was intended to maintain motivating operation (MO; Laraway et al., 2003) control for target items during sessions.

#### 2.6.1. Baseline

All dependent variables (i.e., proximal and distal pointing) were assessed during baseline probe sessions, with each session consisting of five trials. Each baseline sequence consisted of at least three consecutive proximal pointing sessions, followed by distal sessions at 0.61 m, 1.22 m, and 1.83 m, with this rotation repeated until the intervention phase was introduced. Including both proximal and distal baseline sessions allowed for assessment of whether independent points were specific to a single distance (i.e., occurred at either proximal or distal distances, but not both).

Prior to each session block, the experimenter obtained items previously nominated as preferred by each child’s respective clinical team and selected a subset to present in the instructional area. The child’s motivation and preferences for these items were then assessed informally for approximately 10–60 s by observing indicating responses (e.g., reaching toward) and the child’s engagement or non-engagement with the items, consistent with procedures used in prior momentary reinforcer evaluations (e.g., Alcalay et al., 2019; S. L. Morris et al., 2024). Items that elicited positive indicating responses or engagement were included in the session, and preferred items were rotated across sessions and trials to maintain motivation based on ongoing informal preference assessments.

For each trial, the experimenter presented an array of three stimuli, including a target item for which the child had previously indicated preference (e.g., through an indicating response such as a shifting gaze, moving closer, or prior interaction with the item) and two additional items presumed to be neutral or non-preferred. It should be noted that no formal evaluation was conducted to confirm whether the items presumed to be neutral were truly non-preferred. The position of the target stimulus (left, center, or right) was quasi-randomized across trials and sessions to minimize position-biased responding; however, positional bias may have persisted because repeated selection of a given location continued to produce reinforcement (Bourret et al., 2012). We allowed up to five seconds for the participant to emit an indicating response, including pointing, toward a stimulus in the array.

Any pointing that occurred during the baseline phase was recorded and reinforced by providing the participant access to the indicated item. Reinforcing pointing during baseline allowed for evaluation of whether pointing would persist across baseline sessions and whether the intervention was necessary. Participants’ indicating responses that occurred without pointing (e.g., reaching or hand-leading the adult toward an item) were recorded as incorrect on the data sheet, and no programmed reinforcement was provided. This allowed us to assess whether pointing would emerge during baseline due to extinction-induced variability.

#### 2.6.2. Proximal Pointing Treatment

Treatment sessions were conducted identically to baseline sessions (described above), except that following a participant’s indicating response for an item in an array, errorless prompting (Touchette & Howard, 1984) and graduated guidance (Demchak, 1990) were used to prompt a proximal index finger point. Identical to the baseline procedure, each array consisted of one preferred item and two neutral or nonpreferred items (with no formal evaluation of relative preference). Momentary reinforcer analysis procedures (e.g., Alcalay et al., 2019) were implemented continuously across trials. The experimenter monitored participants’ engagement, indicating responses, and interaction with items during and between trials, and items were rotated or replaced within and across sessions if motivation appeared to decrease.

The initial controlling prompt level (Table 1) was identified during the first treatment session based on the least intrusive prompt that evoked a correct proximal index-finger point with 100% accuracy across three consecutive trials. No separate prior assessment was conducted, as doing so could have resulted in inadvertent teaching of the target response before the introduction of the intervention. Of the 12 participants, 10 initially required a full physical prompt. Two participants, Virginia and Gavin, began with a model prompt as their controlling prompt. The intrusiveness of prompts decreased systematically as the experimenter progressed through the graduated guidance hierarchy. Operational definitions for each prompt level are presented in Table 3.

A typical most-to-least prompting sequence proceeded as follows: after the participant demonstrated an indicating response for an item, the experimenter immediately presented the item within an array of three (one preferred, two neutral or nonpreferred) and delivered the identified controlling prompt using errorless prompting to ensure correct responding and establish stimulus control. Prompt fading was implemented systematically within the graduated guidance hierarchy (Table 3) as responding demonstrated stability at a given prompt level. Clinical judgment guided the fading process; for example, when implementing full physical prompts across trials, the experimenter continuously monitored the participant’s movements to determine when less prompting was needed as the child began to independently isolate and extend the index finger. On the subsequent trial, the prompt could be reduced to a partial physical level. Fading from partial physical to model plus partial physical, model-only, and finally independent pointing followed the same process, with prompts adjusted in real time based on the participant’s emerging independence. This procedure supported errorless prompting while facilitating the transfer of stimulus control from prompts to naturally occurring antecedent stimuli. When the participant was able to respond independently, up to 5 s were allowed for responding, consistent with baseline procedures.

During all treatment sessions, when a participant either accepted a prompt to point or independently produced a proximal point, the requested item was delivered immediately. Participants were given access to the delivered item for approximately 30 to 60 s before the next trial. Mastery of proximal pointing was defined as three consecutive sessions (15 total trials) with 100% independent responding. Progression from the intervention phase to the distal pointing generalization phase was contingent upon mastery of independent proximal pointing responses.

Preferred items were delivered following both prompted and independent pointing responses, with no planned differential reinforcement of independent versus prompted points. However, it is possible that participants experienced adventitious differential reinforcement, such that independent points were reinforced more immediately than prompted points, and this may have contributed to the acquisition of the proximal pointing response.

#### 2.6.3. Distal Pointing Generalization

Once mastery criteria were met for the proximal pointing treatment, probe sessions for distal pointing were conducted at 0.61 m, 1.22 m, and 1.83 m. Distal pointing was assessed sequentially from shorter to longer distances, with sessions conducted identically to baseline (described above). If a participant demonstrated ≥80% independent responding (4 out of 5 trials) at one distance, the next greater distance was probed. If independent pointing at any distal distance fell below 80%, the probe was repeated for a minimum of three sessions until the criterion was met. If a participant did not reach 80% independent responding after three sessions at a given distance, treatment for distal pointing at that distance would have been introduced; however, this was not required for any participant (see Section 3).

#### 2.6.4. Maintenance

For each participant, maintenance probes were conducted approximately four weeks after completing the proximal pointing intervention and following the assessment of distal generalization. Procedures were identical to baseline (described above). Maintenance probes included at least one session of proximal pointing and one session for each distal distance at 0.61 m, 1.22 m, and 1.83 m.

## 3. Results

**Figure 1:**
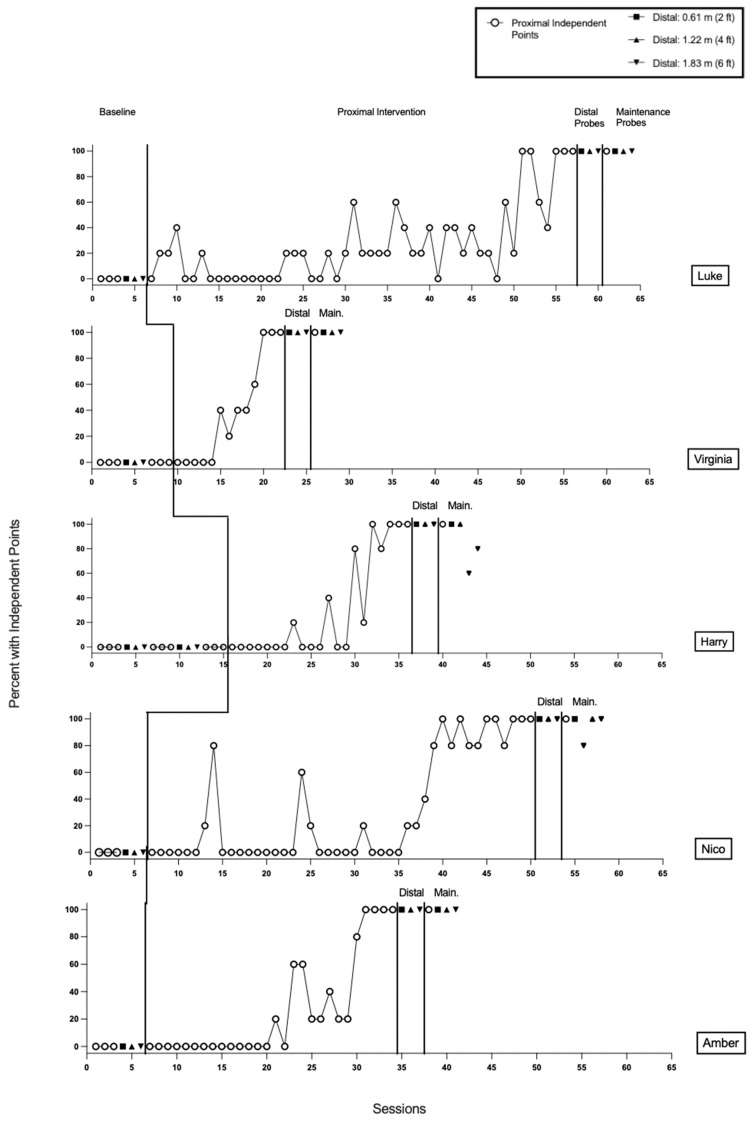
Results for Luke, Virginia, Harry, Nico, and Amber.

**Figure 2:**
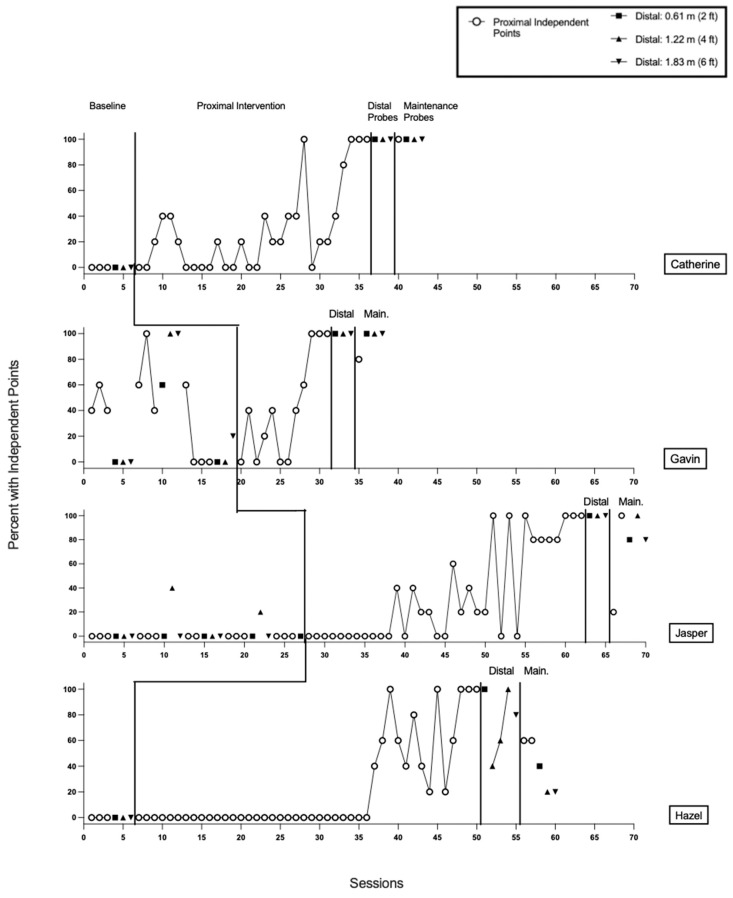
Results for Catherine, Gavin, Jasper, and Hazel.

**Figure 3:**
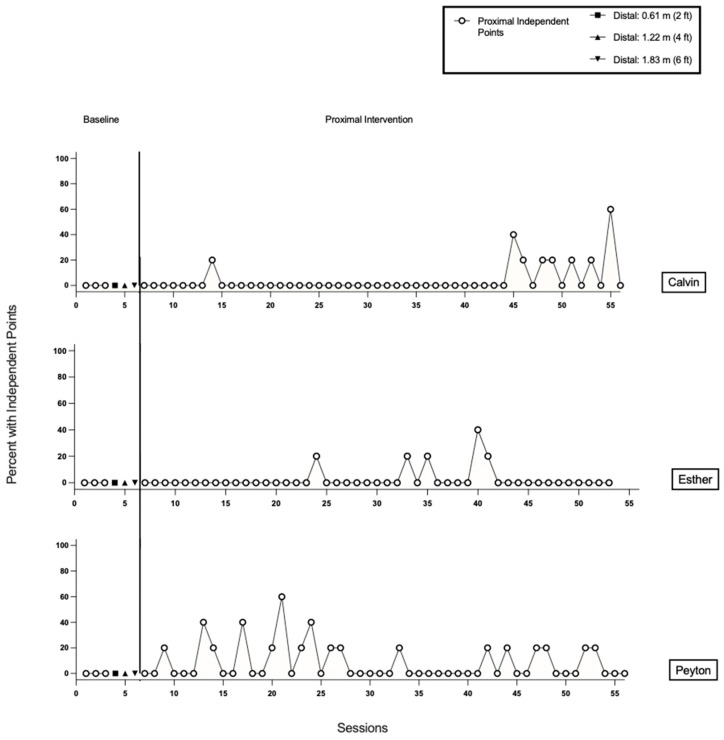
Results for Calvin, Esther, and Peyton.

Results for all participants are presented in Figure 1, Figure 2 and Figure 3. Figure 1 and Figure 2 display data for participants categorized as *responders* to the treatment. *Responders* were participants who demonstrated an individual treatment effect and subsequently met mastery criteria for proximal pointing. Figure 3 displays data for participants categorized as *non-responders* to the treatment. Non-responders were defined as participants who did not achieve mastery of proximal pointing within the predetermined criterion of 50 treatment sessions, and treatment for proximal pointing was discontinued.

### 3.1. Results by Participant

#### 3.1.1. Luke

Results from baseline indicated that Luke did not emit any imperative proximal or distal pointing (Figure 1, Panel 1). Luke required 51 treatment sessions (255 trials) to meet the mastery criterion for proximal pointing. During the generalization phase, Luke emitted pointing with 100% independence across all distal probe sessions at 0.61 m, 1.22 m, and 1.83 m. During the maintenance phase, Luke demonstrated 100% independent pointing across all proximal and distal probes. Luke gave initial assent to 100% of sessions, and no assent withdrawal was observed (Table 4).

#### 3.1.2. Virginia

Results from baseline indicated that Virginia did not emit any imperative proximal or distal pointing (Figure 1, Panel 2). Virginia required 13 treatment sessions (65 trials) to meet the mastery criterion for proximal pointing. During the generalization phase, Virginia emitted pointing with 100% independence across all distal probe sessions at 0.61 m, 1.22 m, and 1.83 m. During the maintenance phase, Virginia demonstrated 100% independent pointing across all proximal and distal probes. Virginia gave initial assent to 100% of sessions, and no assent withdrawal was observed (Table 4).

#### 3.1.3. Harry

Results from baseline indicated that Harry did not emit any imperative proximal or distal pointing (Figure 1, Panel 3). Harry required 21 sessions (105 trials) to meet the mastery criterion for proximal pointing. During the generalization phase, Harry emitted pointing with 100% independence across all distal probe sessions at 0.61 m, 1.22 m, and 1.83 m. During the maintenance phase, Harry demonstrated 100% independent pointing on a proximal probe and distal probes at 0.61 m and 1.22 m. Across two consecutive distal maintenance probes conducted at 1.83 m, Harry demonstrated 60% and 80% accuracy, respectively. Harry gave initial assent to 100% of sessions, and no assent withdrawal was observed (Table 4).

#### 3.1.4. Nico

Results from baseline indicated that Nico did not emit any imperative proximal or distal pointing (Figure 1, Panel 4). Nico required 44 treatment sessions (220 trials) to meet the mastery criterion for proximal pointing. During the generalization phase, Nico emitted pointing with 100% independence across all distal probe sessions at 0.61 m, 1.22 m, and 1.83 m. During the maintenance phase, Nico demonstrated 100% independent pointing a proximal probe and distal probes at 0.61 m and 1.22 m. Across two non-consecutive distal maintenance probes conducted at 1.83 m, Nico demonstrated 80% and 100% accuracy, respectively. Nico gave initial assent to 100% of sessions (Table 4). Assent withdrawal was observed during one session (1.64% of sessions), specifically session 22 of the proximal pointing treatment. For that session, percent accuracy was calculated based on the trials completed (3/5 trials; 60% of the session).

#### 3.1.5. Amber

Results from baseline indicated that Amber did not emit any proximal or distal pointing (Figure 1, Panel 5). Amber required 28 treatment sessions (140 trials) to meet the mastery criterion for proximal pointing. During the generalization phase, Amber emitted pointing with 100% independence across all distal probe sessions at 0.61 m, 1.22 m, and 1.83 m. During the maintenance phase, Amber demonstrated 100% independent pointing across all proximal and distal probes. Amber gave initial assent to 100% of sessions, and no assent withdrawal was observed (Table 4).

#### 3.1.6. Catherine

Results from baseline indicated that Catherine did not emit any imperative proximal or distal pointing (Figure 2, Panel 1). Catherine required 30 treatment sessions (150 trials) to meet the mastery criterion for proximal pointing. During the generalization phase, Catherine emitted pointing with 100% independence across all distal probe sessions at 0.61 m, 1.22 m, and 1.83 m. During the maintenance phase, Catherine demonstrated 100% independent pointing across all proximal and distal probes. Catherine gave initial assent to 100% of sessions, and no assent withdrawal was observed (Table 4).

#### 3.1.7. Gavin

Gavin’s baseline accuracy for imperative pointing was variable across distances (Figure 2, Panel 2). For proximal pointing, accuracy ranged from 0–100% (*M* = 40%). For distal pointing, accuracy ranged 0–60% at 0.61 m (*M* = 20.00%), 0–100% at 1.22 m (*M* = 33.33%), and 0–100% at 1.83 m (*M* = 40.00%). Gavin required 12 treatment sessions (60 trials) to meet the mastery criterion for proximal pointing. During the generalization phase, Gavin emitted pointing with 100% independence across all distal probe sessions at 0.61 m, 1.22 m, and 1.83 m. During the maintenance phase, proximal pointing was maintained at 80% accuracy in one probe session, and distal pointing was maintained at 100% accuracy across probe sessions at 0.61 m, 1.22 m, and 1.83 m. Gavin gave initial assent to 100% of sessions, and no assent withdrawal was observed (Table 4).

#### 3.1.8. Jasper

Results from baseline indicated that Jasper did not emit any proximal pointing or distal pointing at the 0.61 m or 1.83 m distances (Figure 2, Panel 3). However, distal pointing was observed at the 1.22 m distance during two of the four baseline sessions in which that distance was probed. Accuracy for distal pointing on these two probes was 40% and 20%, respectively.

For all participants, baseline sessions were designed to rotate proximal and distal pointing probes, with at least three consecutive proximal pointing sessions before introducing distal probes. For Jasper, a procedural error occurred during session 15, in which a third proximal pointing session was not conducted, and distal pointing probes were reintroduced.

Jasper required 35 treatment sessions (175 trials) to meet the mastery criterion for proximal pointing. During the generalization phase, Jasper emitted pointing with 100% independence across all distal probe sessions at 0.61 m, 1.22 m, and 1.83 m. During the maintenance phase, across two consecutive probes conducted at the proximal distance, Jasper demonstrated 20% and 100% accuracy, respectively. During the maintenance phase, Jasper demonstrated 80% accuracy on probes conducted at the 0.61 m and 1.83 m distances and 100% accuracy on the probe conducted at the 1.22 m distance. Jasper gave initial assent to 100% of sessions, and no assent withdrawal was observed (Table 4).

#### 3.1.9. Hazel

Results from baseline indicated that Hazel did not emit any proximal or distal pointing (Figure 2, Panel 4). Hazel required 44 treatment sessions (220 trials) to meet the mastery criterion for proximal pointing. In the generalization phase, Hazel demonstrated 100% independent distal pointing at 0.61 m. At 1.22 m, accuracy increased across three consecutive probe sessions (40%, 60%, and 100%, respectively), and at 1.83 m, Hazel demonstrated 80% independent responding. During the maintenance phase, proximal pointing was maintained at 60% in each of two consecutive probe sessions. Hazel’s distal pointing accuracy during maintenance was reduced compared to the generalization phase, with 40% at 0.61 m, 20% at 1.22 m, and 20% at 1.83 m. Hazel provided initial assent in 100% of sessions, and no assent withdrawal was observed (Table 4).

#### 3.1.10. Calvin

Results from baseline indicated that Calvin did not emit any proximal or distal pointing (Figure 3, Panel 1). During the treatment phase, 50 sessions (250 trials) were conducted, and Calvin met the predetermined termination criteria for the proximal pointing treatment. Calvin gave initial assent to 100% of sessions, and no assent withdrawal was observed (Table 4).

#### 3.1.11. Esther

Results from baseline indicated that Esther did not emit any proximal or distal pointing (Figure 3, Panel 2). During the treatment phase, 47 sessions (235 trials) were conducted. By session 53, Esther had not demonstrated significant progress with the proximal pointing intervention and had shown no pointing in the last 12 sessions. Given that she was anticipated to meet the predetermined termination criteria, her clinical team decided to discontinue the proximal pointing intervention at that time. Esther provided initial assent for 100% of sessions, and no assent withdrawal was observed (Table 4).

#### 3.1.12. Peyton

Results from baseline indicated that Peyton did not emit any proximal or distal pointing (Figure 3, Panel 3). During the treatment phase, 50 sessions (250 trials) were conducted, and Peyton met the predetermined termination criteria for the proximal pointing treatment. Peyton gave initial assent to 100% of sessions, and no assent withdrawal was observed (Table 4).

## 4. Discussion

### 4.1. Summary of Findings

Across participants, two response patterns emerged. *Responders* to the treatment (*n* = 9) demonstrated acquisition of proximal pointing and subsequently generalized to distal pointing without additional intervention. In contrast, *non-responders* to the treatment (*n* = 3) did not demonstrate acquisition of proximal pointing before reaching the predetermined termination criterion, and the experimental treatment was discontinued.

For all responders, generalization from proximal to distal pointing was observed across 0.61 m, 1.22 m, and 1.83 m distances without additional intervention. Overall, pointing was maintained across participants at the 4-week follow-up, with accuracy ranging from 20% to 100% across distances. When maintenance probes yielded less than 80% accuracy, repeating a probe at the same distance resulted in higher accuracy (e.g., Harry and Nico on 1.83 m distal maintenance probes; Jasper on proximal maintenance probes) except for Hazel.

VB-MAPP scores were used to characterize participants’ social, mand, and vocal repertoires prior to intervention. Altogether, responders and non-responders showed overlapping ranges of overall VB-MAPP scores. Responders were distributed across the full range of scores (10–109.5), and the three non-responders were not clustered exclusively at the lowest end of the score distribution.

Higher or lower baseline VB-MAPP scores were not consistently associated with acquisition of proximal pointing or classification as a treatment responder. For example, some participants with relatively low VB-MAPP scores (e.g., Jasper, Harry) achieved mastery in fewer trials than participants with slightly higher scores (e.g., Nico, Hazel). Gavin, who had the highest VB-MAPP score, achieved mastery in the fewest trials overall. Conversely, some participants with moderate baseline scores (e.g., Calvin, Esther, Peyton) did not acquire proximal pointing within the treatment phase. Together, these findings suggest that although the VB-MAPP is useful for characterizing participants’ repertoires, baseline scores did not strongly predict responsiveness to this particular intervention.

Similarly, the initial controlling prompt level did not appear to predict treatment responsiveness, as participants who began with full physical prompts (e.g., Amber, Hazel) and those who began with less invasive prompts, such as model prompts (i.e., Virginia, Gavin), both demonstrated mastery of proximal pointing and generalized to distal pointing. However, beginning treatment with more invasive controlling prompts may have contributed to longer treatment duration, as participants requiring full physical prompts needed additional sessions to demonstrate mastery of proximal pointing as prompts were systematically faded, compared to those who began with model prompts (e.g., Virginia, Gavin). These preliminary findings suggest that, although the initial controlling prompt level may influence the rate of acquisition, it does not determine whether a participant ultimately responds to the proximal pointing intervention or demonstrates generalization and maintenance of pointing.

### 4.2. Limitations and Implications for Future Research

#### 4.2.1. Experimental Control and Design Considerations

Several limitations related to experimental control should be considered when interpreting the findings of the present study. First, the study was conceptualized as a nonconcurrent multiple baseline design across participants, in which individuals were enrolled and began baseline at different time points. In this arrangement, each participant’s data represents an A–B sequence, and the series of demonstrations across participants serves as the basis for experimental inference. However, because participants were enrolled and assessed at different times without substantial temporal overlap in baseline phases, the data may also be interpreted as a series of A–B demonstrations rather than a fully realized nonconcurrent multiple baseline design. As noted in the single-case design literature, A–B demonstrations provide weaker experimental control than designs that include systematic staggering and temporal overlap across participants (e.g., Cooper et al., 2020; Kazdin, 2019). Future research could strengthen experimental control by employing fully concurrent multiple baseline designs or by presenting participant data in formats that more clearly depict staggered baseline durations across participants and overlapping time points to facilitate visual analysis.

From an experimental control standpoint, the initial delay in observing a treatment effect during the proximal pointing intervention for some participants (i.e., Jasper, Amber, Hazel) may also be considered a limitation. It is possible that the delay and subsequent increase in pointing responses at the proximal distance were influenced by threats to internal validity such as maturation or other extraneous variables.

However, several aspects of the data support the interpretation that the proximal pointing intervention contributed to the observed behavior change. Baseline data across multiple sessions demonstrated stable patterns of minimal or absent pointing prior to intervention. In some cases (e.g., Jasper, Gavin), occasional pointing responses occurred during extended baseline phases; however, these responses did not persist when reinforcement contingencies remained in effect, suggesting that the behavior was not yet under functional stimulus control. Increases in independent pointing occurred only following the introduction of the proximal pointing intervention for all nine participants who acquired the skill, providing replication of treatment effects across individuals.

Additionally, all participants were children with ASD who had significant language delays and limited functional vocal speech. Given these developmental characteristics and the fact that participants had previously received ABA intervention services without demonstrating consistent pointing behavior, it is unlikely that the observed changes reflected maturation alone. Instead, the delayed but subsequent increases in pointing responses likely reflect the acquisition period required for participants to learn the pointing response under relevant establishing operation conditions.

#### 4.2.2. Session Design and Response Variability

Delays in observable treatment effects and variability in response stability, as identified through visual analysis of the data, may be attributable in part to the study design in which each session consisted of five trials. This session length was predetermined to minimize potential participant fatigue and reduce the likelihood of challenging behavior, particularly during baseline, distal probe, and maintenance probe sessions when indicating responses other than pointing did not result in programmed reinforcement. Future research could examine differences in treatment effects under varying session lengths and increased numbers of experimental trials, which may visually demonstrate more rapid treatment response and greater stability across sessions.

#### 4.2.3. Participant Characterization and Assessment Limitations

A limitation of the study is the absence of additional norm-referenced, standardized measures to further characterize participants, particularly regarding cognitive functioning. The present study included historical communicative and language skills via VB-MAPP scores but did not assess participants’ verbal or nonverbal cognitive level, which could have provided additional insight into individual differences in the acquisition of proximal pointing; for example, why three participants did not acquire this skill while nine did. Including cognitive assessments in future studies may allow researchers to examine correlations between cognitive ability and communication outcomes, providing a more comprehensive understanding of factors that may influence skill acquisition for pointing. Additionally, VB-MAPP scores obtained from clinical records did not include measures of IOA or procedural fidelity for administration.

Further, the reinforcer identification procedures used were informal and unsystematic, relying on historical preferences reported by participants’ clinical teams and in-the-moment observation of indicating responses. Written data were not collected regarding whether participants ever reached for presumed neutral or nonpreferred items; however, anecdotally, this did not appear to occur, given the momentary reinforcer evaluation procedures. Nevertheless, variability in the reinforcing efficacy of target stimuli across participants may have contributed to individual differences in intervention outcomes. That is, some participants may not have been as motivated by the items presented, potentially impacting the acquisition of proximal pointing and subsequent distal pointing or maintenance measures. Future studies could consider incorporating systematic preference and reinforcer assessments to support more consistent motivation for target stimuli and more accurately assess the role of reinforcement in response acquisition.

Future research may also include additional characterization measures that include more comprehensive measurements of gesture use, such as the *MacArthur–Bates Communicative Development Inventory: Words and Gestures* (CDI:WG; Marchman et al., 2023), to better capture participants’ baseline gesture production and communication skills prior to intervention. Collecting these data could help identify potential moderators of treatment effectiveness specific to gesture production for children with ASD. Future researchers may also consider administering standardized assessments as pretest and posttest measures to evaluate the effects of interventions targeting gesture use on communication and language development. In the present study, it remains unknown whether teaching pointing led to increases in participants’ early spoken word production or word learning. For minimally verbal children with ASD, understanding these outcomes could be particularly impactful, as such findings may inform the design of targeted interventions to support early language development.

## 5. Conclusions

In conclusion, this study evaluated the efficacy of teaching 12 children with ASD to emit imperative proximal points using a treatment protocol consisting of errorless prompting and graduated guidance. The intervention was effective for nine children, who also demonstrated generalization to distal pointing. Maintenance of proximal and distal pointing at the 4-week follow-up was variable; however, all participants continued to emit proximal and distal points. For three children, mastery of proximal pointing was not demonstrated within the predetermined treatment criterion. Overall, these findings support the notion that autistic children with limited use of gestures can be taught to emit a proximal point to request items, and once learned proximally, distal points are also emitted. Despite these findings, there is still a need for future research to assess the broader impact of teaching pointing on early spoken word production, word learning, and communication development for children with ASD.

## Figures and Tables

**Table 1:** Participant Characteristics.

Participant	Age (Years)	Sex	Race and Ethnicity	VB-MAPP Overall Score (/170)	Social Domain Score (/30)	Mand Domain Score (/25)	Vocal Domain Score (/20)	Initial Controlling Prompt Level
Luke	5	Male	White	44.5	6	5	4.5	Full physical
Virigina	9	Female	NR	46.5	6	5	4	Model
Harry	3	Male	White	12	1	0	1	Full physical
Nico	4	Male	White	13	NR	NR	NR	Full physical
Amber	4	Female	Hispanic or Latino	24.5	4.5	0.5	2	Full physical
Catherine	4	Female	White	15	2	0	2.5	Full physical
Gavin	11	Male	Hispanic or Latino and White	109.5	9.5	7	7	Model
Jasper	5	Male	White	10	2	0	1	Full physical
Hazel	7	Female	NR	30	5	5	3.5	Full physical
Calvin	9	Male	NR	21.5	2	2	2	Full physical
Esther	3	Female	Native Hawaiian/Other Pacific Islander	34	3	4	3	Full physical
Peyton	3	Male	Black or African American	67.5	6	8.5	3	Full physical

Note. VB-MAPP = Verbal Behavior Milestones Assessment and Placement Program (Sundberg, 2008). NR = not reported; exact score not reported in clinical records. Age is each participant’s age at the time of enrollment in the study. Race and ethnicity categories are reported following APA recommendations for demographic reporting (*Publication Manual of the American Psychological Association*, 7th edition) and are based on U.S. Census Bureau classifications (U.S. Census Bureau, n.d.). For Nico, only the VB-MAPP overall score was reported.

**Table 2:** Operational Definitions.

Term	Definition
Proximal index finger pointing	The child refers to an object that is within 0.30 m (1 ft) by touching it or hovering an extended index finger while the other fingers are curled down and separated from the index finger. Use of the index finger to operate a toy or manipulate an item is not considered a proximal point.
Distal index finger pointing	The child refers to a distant object (i.e., more than 0.30 m (1 ft) from the referent) using an extended index finger. The other fingers must be clearly curled down and separated from the index finger.
Assent	The child approaches a location and remains within 1.83 m (6 ft) of it, associated with an activity, and/or uses their communication system, vocal or non-vocal, to indicate “yes” to access the presented activity.
Withdrawal of assent	The child refuses to enter a location, leaves a location (i.e., exits the door or moves away from the area associated with an activity), and/or uses their communication system, vocal or non-vocal, to indicate “no” or reject the activity.

Note. m = meters. ft = foot. Definitions for *proximal index finger pointing* and *distal index finger pointing* adapted from Ramos-Cabo et al. (2021). Definitions for *assent* and *withdrawal of assent* are based on recommendations from C. Morris et al. (2021, 2024), and Breaux and Smith (2023).

**Table 3:** Prompt Level Descriptions.

Prompt Type	Prompt
Full physical	Adult provides full hand-over-hand physical guidance, isolating the child’s index finger and physically lifting the arm, wrist, or hand to point toward the item.
Partial physical	Adult guides part of the child’s upper limb to initiate a pointing gesture with index finger isolation. This may include hand-over-wrist, hand-over-arm, or hand-over-elbow guidance, and/or a shoulder tap to prompt forward arm movement.
Model + Partial physical	Adult models the pointing gesture with index finger isolation while simultaneously delivering a partial physical prompt (e.g., light guidance on wrist or elbow).
Model	Adult models a pointing gesture with index finger isolation to prompt imitation.
Independent	The child initiates and completes the pointing gesture without any physical or modeled prompts from the adult.

**Table 4:** Assent and Assent Withdrawal Measures.

Participant	Initial Assent (% of Sessions)	Assent Withdrawal (% of Sessions)
Luke	100	0
Virginia	100	0
Harry	100	0
Nico	100	1.64
Amber	100	0
Catherine	100	0
Gavin	100	0
Jasper	100	0
Hazel	100	0
Calvin	100	0
Esther	100	0
Peyton	100	0
Mean ± SD	100 ± 0	0.92 ± 2.8

Note. Percentages represent the proportion of sessions in which initial assent or assent withdrawal was observed. SD = standard deviation.

## Data Availability

Data are contained within the article and are available from the first author upon reasonable request.

## Note

1 Individuals and communities may prefer person-first or identity-first language. This article uses both approaches to reflect and respect those preferences.

## Supplementary Materials

The following supporting information can be downloaded at: https://www.mdpi.com/article/10.3390/bs16030401/s1, Figure S1: Session Data Sheet; Figure S2: Session Treatment Integrity Checklist.

## Abbreviations

The following abbreviations are used in this manuscript:

ABA	applied behavior analysis
ASD	autism spectrum disorder
BCBA	board-certified behavior analyst
CDI: WG	MacArthur–Bates Communicative Development Inventory: Words and Gestures
EO	establishing operation
ft	feet
IOA	interobserver agreement
JA	joint attention
m	meters
M	mean
MO	motivating operation
NR	not reported
TD	typically developing
TI	treatment integrity
VB-MAPP	Verbal Behavior Milestones Assessment and Placement Program
