# Supplementary material for: Improving Use of Social Communicative Gestures by Children with Autism
Source: Behav Sci (Basel). 2026 Mar 10;16(3):401. doi: 10.3390/bs16030401 (PMC13024087; doi:10.3390/bs16030401)
Supplement: Supplementary file 1 [file behavsci-16-00401-s001.zip › behavsci-4116067-supplementary.pdf]

Supplemental Material

Figure S1

Session Data Sheet

Primary or Reliability Data: ______________________

Session #:

Date: __________ Data Collector: ____________ Implementor: _____________Condition: ______________

Circle: Proximal Distal – 2 ft. Distal – 4 ft. Distal – 6 ft.

Assent to and throughout session: Yes No (note when withdrawal observed): ______________________

#	Target	Array	IND	FP	PP	M+PP	M
1		L M R	+ E NR	+ E NR	+ E NR	+ E NR	+ E NR
2		L M R	+ E NR	+ E NR	+ E NR	+ E NR	+ E NR
3		L M R	+ E NR	+ E NR	+ E NR	+ E NR	+ E NR
4		L M R	+ E NR	+ E NR	+ E NR	+ E NR	+ E NR
5		L M R	+ E NR	+ E NR	+ E NR	+ E NR	+ E NR

Figure S2

Session Treatment Integrity Checklist

Session #: **Live or Video** (Circle one)

Date: __________ Data Collector: ____________ Implementor: _____________Condition:

____

Circle: Proximal Distal – 2 ft. Distal – 4 ft. Distal – 6 ft.

#	Steps Observed	+/-
1	Pairs with SR+ to begin session.	
2	Assesses MOs before initiating a trial	
3	Presents an array of 2-3 items at least a foot apart to initiate trial	
4	During teaching trials, immediately proves a prompt after an IR	
5	Uses appropriate controlling prompt during teaching trials	
6	Provides praises and the preferred item after correct responses.	
7	During baseline sessions, space out trials for 30 seconds – 1 minute.	
8	Allows exit if assent withdrawn (mark n/a if not withdrawn).	
	Total Percent:	
